# Advances in efficacy prediction and monitoring of neoadjuvant immunotherapy for non-small cell lung cancer

**DOI:** 10.3389/fonc.2023.1145128

**Published:** 2023-05-17

**Authors:** Yunzhen Wang, Sha Huang, Xiangwei Feng, Wangjue Xu, Raojun Luo, Ziyi Zhu, Qingxin Zeng, Zhengfu He

**Affiliations:** ^1^ Department of Thoracic Surgery, Sir Run Run Shaw Hospital, Zhejiang University School of Medicine, Hangzhou, China; ^2^ Department of Thoracic Surgery, Longyou County People’s Hospital, Longyou, China

**Keywords:** non-small cell lung cancer, neoadjuvant immunotherapy, chemo-immunotherapy, efficacy prediction, biomarkers

## Abstract

The use of immune checkpoint inhibitors (ICIs) has become mainstream in the treatment of non-small cell lung cancer (NSCLC). The idea of harnessing the immune system to fight cancer is fast developing. Neoadjuvant treatment in NSCLC is undergoing unprecedented change. Chemo-immunotherapy combinations not only seem to achieve population-wide treating coverage irrespective of PD-L1 expression but also enable achieving a pathological complete response (pCR). Despite these recent advancements in neoadjuvant chemo-immunotherapy, not all patients respond favorably to treatment with ICIs plus chemo and may even suffer from severe immune-related adverse effects (irAEs). Similar to selection for target therapy, identifying patients most likely to benefit from chemo-immunotherapy may be valuable. Recently, several prognostic and predictive factors associated with the efficacy of neoadjuvant immunotherapy in NSCLC, such as tumor-intrinsic biomarkers, tumor microenvironment biomarkers, liquid biopsies, microbiota, metabolic profiles, and clinical characteristics, have been described. However, a specific and sensitive biomarker remains to be identified. Recently, the construction of prediction models for ICI therapy using novel tools, such as multi-omics factors, proteomic tests, host immune classifiers, and machine learning algorithms, has gained attention. In this review, we provide a comprehensive overview of the different positive prognostic and predictive factors in treating preoperative patients with ICIs, highlight the recent advances made in the efficacy prediction of neoadjuvant immunotherapy, and provide an outlook for joint predictors.

## 1 Introduction

Lung cancer has one of the highest incidence rates worldwide and is responsible for the most deaths (1). Non-small cell lung cancer (NSCLC) is a major type of lung cancer accounting for approximately 85% of lung cancer cases; only 30%–40% of patients diagnosed with NSCLC present with resectable disease (2, 3). Surgical resection is the cornerstone for the treatment of early-stage NSCLC and is also one of the most effective means for the treatment of stage IIIA disease for attaining resectable status by neoadjuvant chemotherapy (4–6). Preoperative chemotherapy improves both progression-free survival (PFS) and overall survival (OS); however, it improves the 5-year survival rate by only 5% and fails to meet clinical needs (7). Recently, activating the human immune system to fight cancer, including NSCLC, by blocking inhibitory immune checkpoints has gained attention. Several phase III trials have confirmed the role of pembrolizumab, an immune checkpoint inhibitor (ICI), as a standard first-line treatment for patients with locally advanced or metastatic NSCLC (8–10). Notably, compared with monotherapy, ICIs plus platinum-based chemotherapy resulted in significantly longer OS and PFS regardless of PD-L1 expression (11–14).

Given the effectiveness of immunotherapy, an increasing number of clinical trials evaluating these agents are rapidly moving from advanced NSCLC to earlier stages of the disease (15). These trials have shown a high percentage of patients with resectable NSCLC achieving a major pathological response (MPR) of up to 86% and a pathological complete response (pCR) of approximately 9%–63% (16, 17). These data suggest the feasibility and efficacy of neoadjuvant immunotherapy in tumor down-staging in patients without increasing the incidence of adverse effects or surgical delay (18). In early 2022, National Comprehensive Cancer Network (NCCN) updated its guidelines for neoadjuvant systemic therapy according to the result of CheckMate 816 presented at the 2021 AACR annual meeting (19). The regimen of nivolumab plus platinum-doublet chemotherapy was first added to the guidelines as neoadjuvant systemic therapy. The preliminary efficacy of chemo-immunotherapy in NSCLC patients is being widely discussed and has prompted research worldwide (15–18). Based on the limited data currently available, as many as 17 different clinical trials about neoadjuvant immunotherapy are currently registered in ClinicalTrials.gov (20). These landmark clinical trials of neoadjuvant immunotherapy have ushered in a new era in NSCLC therapy.

Regardless of the benefits of neoadjuvant immunotherapy, not all patients will experience favorable responses to treatment. Different clinical trials reveal wide efficacy gaps (17). There exists a significant population of patients who do not sufficiently respond to ICIs and may even suffer severe immune-related adverse effects (irAEs) or hyperprogression (21). The heterogeneous objective response rate (ORR) in NSCLC patients who received immunotherapy indicated that the selection of effective biomarkers to better stratify patients for immunotherapy is important. Preliminary data generated by clinical trials for advanced NSCLC patients treated with ICIs have disclosed some biomarkers that are associated with response to immunotherapy (22). These can be roughly divided into the following four categories (23): 1) tumor-intrinsic biomarkers, including programmed cell death ligand 1 (PD-L1), tumor mutational burden (TMB), and specific gene alterations; 2) tumor microenvironment biomarkers, including tumor-associated immune cells (TAICs), and T-cell receptor (TCR) repertoire; 3) liquid biopsies, including peripheral blood cells and circulating tumor DNA (ctDNA); 4) host-related biomarkers, including clinical characteristics, sex, and human leukocyte antigen-1 (HLA-I).

The study of predictive factors associated with the efficacy of neoadjuvant immunotherapy is still in its infancy, and most explorations of neoadjuvant-related biomarkers are built upon existing biomarkers that have been identified in studies on ICI monotherapy. However, factors underlying the therapeutic efficacy of combination therapy and monotherapy may differ and, thus, cannot be generalized. Due to its short therapeutic cycles, periodic image reviewing, and definitive assessment of pathological responses, neoadjuvant immunotherapy is an ideal pattern for research biomarkers. This pattern can provide more opportunities to observe and assess biological changes at different times of neoadjuvant immunotherapy.

In this review, we summarize the positive prognostic and predictive factors that have been used to predict the efficacy of neoadjuvant immunotherapy in NSCLC patients, as well as the recent advances in the development of biomarkers that can be used to better facilitate patient selection.

## 2 Controversial biomarkers for predicting neoadjuvant immunotherapy efficacy

### 2.1 PD-L1 and tumor mutational burden

PD-L1 is a key protein in the advancement and development of immunotherapy. PD-L1 expression assessed by immunohistochemistry (IHC) was the first Food and Drug Administration (FDA)-approved companion or complementary diagnostic test for ICI monotherapy in NSCLC patients (24, 25). Owing to data from several studies, nivolumab was approved for NSCLC patients irrespective of the PD-L1 status (26).

Currently, PD-L1 has been investigated as a biomarker in several clinical trials of neoadjuvant immunotherapy. The phase 2 Lung Cancer Mutation Consortium 3 (LCMC3) trial showed that MPR was associated with baseline PD-L1 tumor proportion score (TPS) in NSCLC patients treated with atezolizumab monotherapy; a considerably higher pathological response was observed in patients with TPS ≥ 50% compared with those with TPS < 50% (27, 28). In CheckMate 816 trial, a greater event-free survival (EFS) and pCR benefit with nivolumab plus chemotherapy were seen across subgroups of a tumor PD-L1 expression level of over 1% (29). The NEOSTAR trial showed that those who achieved MPR or high radiographic response had higher PD-L1 expression (30). A similar result was seen in another trial of nivolumab plus ipilimumab in patients under the same dose and interval therapy (31).

In contrast, correlative data from some other trials of neoadjuvant immunotherapy did not show a relationship between PD-L1 status and the clinical benefits of NSCLC patients. Forde et al. and Altorki et al. found no association between PD-L1 tumor status and MPR (32, 33). Interestingly, when NSCLC patients were treated with typical ICIs like atezolizumab or nivolumab combined with paclitaxel and carboplatin, tumor PD-L1 expression tended to show no correlation with pathological response (34, 35). These results are in contrast to those of early studies on monotherapy (28, 30).

TMB, as a genetic characteristic of tumorous tissue, is emerging as a potential predictive biomarker of response to ICIs. TMB could be processed to neo-antigens, and higher TMB resulted in more effective T-cell recognition, which is correlated with better ICI outcomes (36). Although the US FDA approved pembrolizumab for TMB-high solid tumors, including unresectable NSCLC in 2020, there are both pros and cons to the clinical utility of TMB in neoadjuvant immunotherapy (37, 38). Previous trials, including the KEYNOTE-021, 189, and 407, have shown that TMB is not associated with the efficacy of chemo-immunotherapy, suggesting that treatment with ICIs combined with chemo-agents may confound the application of TMB (36, 39). Similarly, the LCMC3, NEOSTAR, and NADIM trials revealed that TMB was not significantly associated with MPR or patient survival (28, 31, 35). The CheckMate 816 trial also incorporated TMB into analyses and found that pCR benefit was seen with nivolumab plus chemotherapy regardless of TMB value (29). Only in patients in the NCT02259621 trial were a high mean of TMB predicted MPR and the mutation-associated neoantigen burden associated with pathological response (32); however, there was no significant correlation between TMB and PD-L1 expression (32).

### 2.2 Specific gene alterations

NSCLC with epidermal growth factor receptor (EGFR) mutation, anaplastic lymphoma kinase rearrangements, or ROS1 mutations is currently recognized as a negative predictor of immunotherapy efficacy (40, 41). NSCLC patients harboring EGFR mutations benefit less from ICI treatment despite high PD-L1 expression (42). Several studies have shown that PD-L1 expression is regulated by complex mechanisms, including EGFR. Chen et al. found that EGFR activation upregulated PD-L1 via phosphorylating ERK and c-Jun pathway (43). Data from experimental studies showed that EGFR mutations could also upregulate PD-L1 through a variety of pathways, including NF-kB, YAP, JAK/STAT, and PI3K/AKT/mTOR (44, 45). Therefore, EGFR mutation may cause immune escape through the upregulation of PD-L1 expression. Moreover, EGFR mutation may also influence immune cell infiltration. EGFR mutational activation might reduce the MHC-I expression through the ERK-MEK pathway, resulting in a decreased number of infiltrating CD8^+^ T cells, which may then contribute to the poor response to ICIs (46). However, the application of EGFR as a negative biomarker to predict the efficacy of immunotherapy remains controversial, and the underlying mechanisms and interactions with ICI therapy are complex (47). Different benefits of ICI therapy could be observed in different subtypes of EGFR mutations. Patients with EGFR L858R had better benefits from immunotherapy than those with 19Del (40).

In contrast, patients with KRAS mutations or BRAF V600E mutations might have a higher response to ICIs (48). These patients were found to have increased PD-L1 expression and high TMB burden (49). The potential mechanism may involve that RAS mutations can stabilize the mRNA encoding the PD-L1 protein through downstream signals; thus, tumor cells continue to synthesize PD-L1. There are limited data on the impact of oncogenic driver genes on the response to immunotherapy in patients with early-stage NSCLC since most clinical trials have excluded patients with tumors with mutations in the oncogenic genes.

The cohort in the LCMC3 trial observed that patients without STK11/LKB1 mutations or Keap1 mutations more frequently achieved MPR (27). Moreover, the most recent data published from the LCMC3 trial showed that co-mutant STK11 and KRAS portended worse pathological responses (28). Significantly, NSCLC patients with tumors with mutant STK11 not only have no radiographic or pathological response (34) but also suffered progressive disease when STK11 is co-mutated with KRAS (31). Tumor intrinsic pathways including STK11/LKB1 and KEAP1 are associated with non-T cell-inflamed tumor microenvironment (TME), which is also called a “cold” tumor, thus impairing the clinical efficacy of immunotherapy (50). Inactivation of STK11 signaling stimulates cancer cells to produce G-CSF, CXCL7, IL-6, and IL-1β, thereby recruiting tumor-associated neutrophils, which results in suppression of cytotoxic T-cell activity (51, 52). While all of these results have been mutually verified in different studies, conclusions are limited given the small number of patients in each clinical trial. Further clinical trials that include large panels of specific gene alterations will help investigate novel therapies to overcome STK11 mutation-mediated resistance to neoadjuvant immunotherapy.

### 2.3 Blood parameters and host-related markers

Blood parameters represent attractive biomarkers because blood is easily accessible and can be analyzed repeatedly over time. Some metrics and ratios of complete blood count (CBC) have been suggested as markers for predicting the efficacy of ICIs and patient outcomes. In studies on advanced NSCLC patients, Diem et al. and Ren et al. reported that high values of neutrophil-to-lymphocyte ratio (NLR) and platelet-to-lymphocyte ratio (PLR) before treatment are prognostic markers significantly correlating with poor survival and lower response rates in patients treated with nivolumab monotherapy (53, 54). NADIM trials incorporated NLR and PLR to study the association of these parameters with the degree of pathological response and found that only decreased PLR after neoadjuvant treatment was associated with pCR (55). Moreover, lactate dehydrogenase (LDH) or peripheral blood tumor marker carcinoembryonic antigen (CEA) might also be a reliable biomarker to predict immunotherapy efficacy in NSCLC patients (56–58). However, chemo-agents used in neoadjuvant immunotherapy may affect patients’ blood parameters, making the applicability of blood-related biomarkers uncertain.

Host-related markers contain various factors about the patients’ clinical characteristics, such as sex, age, body mass index, smoking, personal history, and HLA complex (59–62). Several studies have shown that the efficacy of ICI monotherapy is better in men than in women, even in the case of high PD-L1 expression NSCLC (62, 63). In contrast, women benefit significantly more from ICIs plus chemotherapy than men with advanced lung cancer (64, 65). The sex-based difference in antitumor immune response relies on a complex interplay between immune evasion mechanisms, hormones, genes, and behavioral factors (66, 67). In CheckMate 816 trial, both male and female patients benefitted more from nivolumab plus chemotherapy over chemotherapy alone. Interestingly, median EFS was longer in women than in men in both arms (29). Additionally, elder patients may achieve poor immunotherapy efficacy due to immunosenescence. Whether the benefit of immunotherapy is age-dependent remains controversial (68–71). Further clinical research should take host-related factors into account to eliminate bias, explore potential mechanisms, and stratify populations that benefit from neoadjuvant immunotherapy.

## 3 Potential biomarkers for predicting neoadjuvant immunotherapy efficacy

### 3.1 Tumor-associated immune cells and tertiary lymphoid structures

The unique advantage of neoadjuvant therapy compared with other advanced NSCLC treatments is that pre- and post-neoadjuvant immunotherapy tissue specimens can be obtained during the whole treatment cycle. The different types of tumor-infiltrating lymphocytes (TILs) in the TME can be used to predict the prognosis and response to immunotherapy (72). TILs, as part of tumor-associated immune cells, can be assessed in tumor tissues using immunohistochemistry or other high-plex multiplex immunofluorescence.

The NEOSTAR trial analyzed immune profiling of resected tumor tissues and found that CD3^+^ TILs, CD3^+^CD8^+^ TILs, and CD3^+^CD8^+^CD45RO^+^ memory TILs were significantly higher in tumors treated with nivolumab + ipilimumab than in those treated with monotherapy. However, these increases were irrespective of MPR (30). Immunologic analyses from the LCMC3 trial revealed that lower frequencies of ILT2^+^NKG2A^+^ and ILT2^+^NKG2A NK cells, and ILT2^+^ NK-like T cells were strongly associated with MPR in NSCLC patients (73). The pCR patients from the NADIM trial had a higher percentage of CD3^+^ CD4^+^ PD-1^+^ cells at diagnosis than non-pCR patients (74). The validity of this cell subset as a predictive marker had also been verified, with an area under the curve (AUC) of 0.728. Furthermore, patients with complete pathologic response (cPR) also had higher levels of NKG2D expression on CD56^+^ T cells, CD25 expression on CD4^+^CD25hi^+^ cells, and CD69 expression on intermediate monocytes when compared to non-cPR patients (74). The above studies indicate that the different types of TILs could predict the response to neoadjuvant immunotherapy to some extent.

The TME not only plays an important role in TILs and mediates the initiation and progression of tumors but also participates in the aggregation of immune cells that developed in non-lymphoid tissues at the tumor site (75). These organized cellular aggregates, composed of B cells, T cells, dendritic cells, and high endothelial venules are called tertiary lymphoid structures (TLSs). Several studies have shown that the presence of TLSs is associated with favorable responses and prognosis to immunotherapy in most solid tumors including NSCLC (76, 77). Cottrell et al. assessed the specific immunologic features of TLS in NSCLC patients treated with neoadjuvant nivolumab using quantitative immune-related pathological response criteria and demonstrated that TLSs are important in the antitumor immune response in pCR and MPR patients (78). Large-scale studies are still needed to understand the complex relationship between immune cell profiles and patient outcomes.

### 3.2 T-cell receptor sequencing

TCR is a unique protein complex found on the surface of T cells that is responsible for recognizing fragments of antigens, including tumor neoantigens (79). Emerging evidence has indicated that TCR sequencing could be used as a dynamic biomarker of ICI response (80). Chemotherapy is the cornerstone of neoadjuvant immunotherapy. Chemo-agents have the great capability of tumor debulking and releasing neoantigens while killing cancer cells. The interplay between neoantigens and TCR plays a critical role in tumor-specific T cell-mediated antitumor immune response (81).

A trial of neoadjuvant administration of nivolumab monotherapy in patients with early-stage lung cancer revealed that MPR patients had a higher frequency of T-cell clones in both the tumor and peripheral blood than non-MPR patients (32). One patient’s cPR neoantigen-specific T-cell clones rapidly increased in peripheral blood and were maintained for up to 4 weeks after treatment (32). In a study using samples obtained from the same clinical trial (NCT02259621), Caushi et al. found that some specific T-cell clonotypes for mutation-associated neoantigens (MANAs) were expanded and detected in MPR patients, suggesting that there were differences between MANA-specific TIL in ICI-responsive versus ICI-resistant NSCLC (82).

With the development of high-throughput sequencing technology, the TCR repertoire can be assessed through various features, including density, diversity, and clonality. In one exploratory analysis of the NADIM trial, next-generation TCR sequencing was performed using pre-treatment and post-treatment peripheral blood and tissues obtained from NSCLC patients (83). Baseline tissue TCR unevenness was associated with cPR to neoadjuvant chemo-immunotherapy. Moreover, compared with TPS (AUC of 0.767) and TMB (AUC of 0.550) as biomarkers, the top 1% clonal space of TCR achieved a higher diagnostic potential, with an AUC of 0.967, to identify cPR patients (83). The TCR repertoire showed good performance in predicting response to neoadjuvant immunotherapy. Further studies are warranted in larger cohorts to precisely identify specific TCR repertoire.

### 3.3 Circulating tumor DNA

CtDNAs are short DNA fragments released from tumors into peripheral blood and can be quantified in liquid biopsies to predict tumor recurrence (84). The detection and sequencing of ctDNA may reveal minimal residual disease (MRD) and identify NSCLC patients who are at high risk of recurrence (85–88).

One large-scale, multicenter prospective cohort study showed that ctDNA-MRD positivity was an independent risk factor for shortened recurrence‐free survival in lung cancer surgery patients (89). The NADIM trial evaluated ctDNA levels before and after neoadjuvant treatment and found that patients with low ctDNA levels at baseline had significantly improved PFS and OS than those with high ctDNA levels; moreover, patients with undetectable ctDNA after treatment were significantly associated with long PFS and OS (35, 90). In CheckMate 816 trial, a higher percentage of patients showed ctDNA clearance with chemo-immunotherapy than with chemotherapy alone, and these patients had longer EFS than those without ctDNA clearance (29). Moreover, ctDNA is correlated with cPR (29). Although there are limited data on the predictive power of ctDNA for assessing neoadjuvant chemo-immunotherapy efficacy, several clinical trials have revealed that ctDNA is a potential biomarker for predicting patients’ survival outcomes. Dynamic ctDNA monitoring may be useful for designing new clinical trials.

### 3.4 Gut microbiota

Gut microbiota is also one of the currently investigated biomarker objects, which can modulate the host immune system and maintain tissue homeostasis (91). Accumulating evidence seems to show the gut microbiota as a potential diagnostic tool to predict response or resistance to ICIs through its extensive influence on local and systemic immune systems (92). Gut microbiota has been assessed in the NEOSTAR trial using targeted 16S ribosomal RNA gene sequencing to explore the link between MPR status and the composition of gut microbiota. *Paraprevotella* and *Akkermansia* spp. were associated with MPR in neoadjuvant patients, and *Dialister* sp. was associated with a decrease in nivolumab toxicity (30). The gut microbiota can alter the efficacy and toxicity of ICI agents. Currently, the NADIM study and other trials are also exploring gut microbiota.

## 4 Cutting-edge progress in biomarker exploration

### 4.1 Multi-omics

With the rapid development of omic methods (genomics, proteomics, transcriptomics, metabolomics), massive omics data have become available for clinical analysis (93). Rich et al. described a blood-based host immune classifier (HIC) proteomic testing to classify NSCLC patients. HIC classification could predict survival with ICI-based therapy and select NSCLC patients who were responding to immunotherapy (94). Zhang et al. integrated multi-omics analysis in one NSCLC patient after neoadjuvant immunotherapy and revealed that specific genomic phenotypes and lower immunogenicity were attributed to an inferior immunotherapy efficacy (95). Different omics or diverse genomic phenotypes could be influenced by each other. Some unique homologous recombination deficiency events in combination with TMB, TME, or various intratumor heterogeneity ultimately influence the therapeutic outcomes of neoadjuvant immunotherapy in NSCLC patients (96). Multi-omics data can identify different molecular subtypes associated with different prognoses in NSCLC patients treated with ICIs (97). There is still a lack of research and clinical trials to standardize the acquisition of and analysis tools for omics data in neoadjuvant immunotherapy. Further, neoadjuvant-related studies should investigate the potential applications of omics tools in chemo-immunotherapy.

### 4.2 Image features

Tumor computational imaging can extract a wealth of information about the entire tumor burden, cancer lesion, and para-carcinoma tissues, which may reflect immune response in NSCLC. Khorrami et al. developed a unique feature set called delta-radiomic analysis (DelRADx), which identified the changes in the radiomic texture of CT patterns from the intra- and peri-tumoral regions before and after immunotherapy. Using DelRADx features, the model achieved an AUC of 0.88 in distinguishing responders from non-responder to immunotherapy and was also associated with OS in NSCLC (98). Yang et al. utilized PET-CT to investigate the correlation between radiological, metabolic (^18^F-FDG), and pathological responses in lung cancer patients who underwent neoadjuvant immunotherapy plus surgery (99). The ^18^F-FDG-reflected metabolic activity revealed the presence of invaded tumor-draining lymph nodes (TDLNs) that are associated with poor pathological responses. Their work showed the potential utility of PET-CT in predicting the pathological response to ICIs (99). Recently, with the help of machine learning algorithms, computational imaging has achieved impressive successes in stratifying and quantifying the radiomic features of NSCLC. As will be detailed in the following sections, machine learning algorithms show powerful prediction performance.

### 4.3 Machine learning

Machine learning (ML) is a branch of artificial intelligence (AI) involving algorithms that can be trained to make predictions by analyzing data. Although there is a lack of prospective studies on the application of ML in predicting neoadjuvant efficacy, some studies have made impressive attempts to build prediction models by combining machine learning and various data.

AI algorithms can automatically quantify radiographic characteristics from CT data of NSCLC patients. AI-based characterization of lung cancer lesions can be used as non-invasive radiomic biomarkers, which had a good predictive ability for predicting good immunotherapy response in advanced NSCLC patients receiving immunotherapy and could also predict OS with an AUC of up to 0.76 (100). Yoo et al. constructed a high-performance ML model with AUC over 0.97 from ^18^F-FDG PET-CT radiomics features to predict pCR after neoadjuvant chemo-immunotherapy in NSCLC (101). The accuracy of the prediction using the ML model was significantly higher than that derived using conventional image features (101, 102).

Several types of omics data, such as RNA expression levels, immune-related gene panels, and immune-related biomarkers, from peripheral blood samples or tumor tissues of NSCLC patients treated with ICIs, could be combined with bioinformatics and ML techniques to improve the predictive performance of the model (103, 104). ML has also been applied in some clinical trials. In exploratory analyses of the LCMC3 trial, more than 100 pre-treatment blood samples were used to construct a predictive model for MPR by evaluating immune cell subsets. The final multiparametric model was significantly correlated with MPR with an AUC in the test set of 0.726 (28). Wu et al. integrated data from eight atezolizumab clinical trials to construct a mortality prediction model using different ML algorithms (105). The results showed that random forest (RF) with an AUC of 0.844 reached the highest performance in prediction tasks. Similarly, Benzekry et al. found that RF (AUC of 0.74) was the best ML model to predict disease control rate using NSCLC patients’ simple clinical and hematological data (106). ML algorithms are good at handling non-linear problems and massive calculations. Prelaj et al. integrated real-world data and the blood microRNA signature classifier to develop a new predictive model of ICI response in NSCLC (107). Vanguri et al. developed a dynamic deep attention-based multiple-instance learning model with masking (DyAM) using a cohort of 247 NSCLC patients with multimodal data including radiological, histopathologic, and genomic features and known outcomes to immunotherapy (108). However, these high-volume data did not help much in performance boosting. The AUC value of these ML-based models was between 0.8 and 0.87.

## 5 Discussion

Neoadjuvant immunotherapy has gradually become a mainstay in the treatment of NSCLC. The promising results of neoadjuvant immunotherapy in resectable NSCLC have been confirmed by several prospective randomized controlled trials. While some patients respond to chemo-immunotherapy, a considerable proportion of NSCLC populations fail to benefit from it. It seems all the more necessary today to discover and develop reliable biomarkers for efficacy prediction when chemo-immunotherapy seems to achieve population-wide treating coverage irrespective of PD-L1 expression.

**Figure 1:**
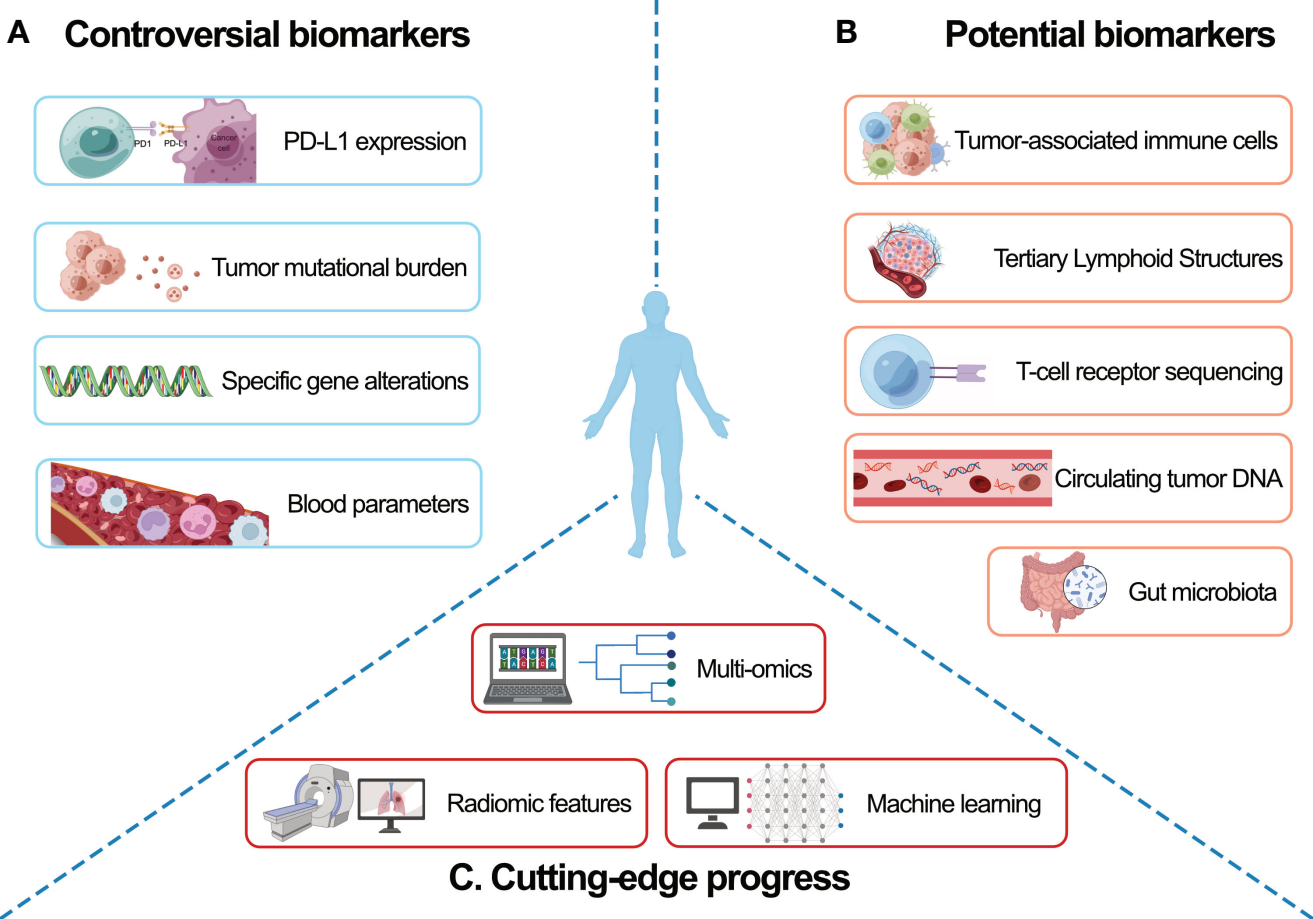
Summary of biomarkers for predicting neoadjuvant immunotherapy efficacy in NSCLC. Biomarkers were grouped by predictive ability: **(A)** controversial biomarkers, **(B)** potential biomarkers, and **(C)** cutting-edge progress in biomarker exploration. Graph drawing created with BioRender.com and Figdraw (https://www.figdraw.com/static/index.html, access date 14 Jan 2023). NSCLC, non-small cell lung cancer.

Based on previous and ongoing clinical trials, we summarized the biomarkers that benefit the prediction of neoadjuvant immunotherapy efficacy and highlight the potential biomarkers for efficacy prediction; we envision the cutting-edge progress made in improving the performance of biomarker-related models (**Figure 1**). These advances in biomarker-directed therapy have led to improvements in OS.

The accuracy of tumor-intrinsic biomarkers such as PD-L1 and TMB has decreased with the addition of chemotherapeutic regimens in NSCLC neoadjuvant immunotherapy that are mutagenic and may thus induce a higher TMB score. The cutoff for TMB evaluation to select NSCLC patients for treatment with ICIs is still currently controversial and may be due to differences in tumor biology and the microenvironment. It is still not routinely used in clinical practice due to the poor reproducibility of TMB results. Therefore, different clinical trials gave different cutoff TMB values (28, 31, 35). PD-L1 is key in immunotherapy, while various posttranslational modifications of PD-L1 protein may affect clinical detection and treatment efficacy. Lee et al. found that heavy glycosylation of PD-L1 could lead to false-negative readouts in clinical bioassays, and deglycosylated PD-L1 is a more reliable biomarker to guide immunotherapy (109). With the widespread application of immunotherapy, the bioassays of tumor-intrinsic biomarkers should also be revolutionized.

With the continuous development of immunofluorescence, flow cytometry, and next-generation sequencing technology, new kinds of potential biomarkers such as TILs, TLS, TCR, or ctDNA are being recognized. Nevertheless, it is difficult to standardize a cutoff for those biomarkers. There is a drift between statistical findings and clinical applications due to the lack of prospective validation studies. Further clinical trials should incorporate AUC, C-index, and calibration to validate the predictive efficacy of these biomarkers. The cost of bioassays is equally of concern. Enrolling high-volume multi-omics data and modifying ML algorithms can improve the performance of prediction tasks. Most data are obtained from retrospective studies, and further studies should consider randomized clinical trials, prospective cohorts, or real-world data for inclusion.

First-line neoadjuvant chemo-immunotherapy is starting to and will revolutionize the current paradigm of resectable NSCLC treatment. Identifying biomarkers for chemotherapy or immunity may be more difficult in combination models. The interaction and crosstalk within chemo-agents and ICIs make it more variable to explore. The advantage of exploring biomarkers under neoadjuvant therapy is that it can better combine the association between dynamic biomarkers in pre- and post-treatment and patient outcomes. Selecting pCR patients or long-term survival patients to further analyze the key factors benefitting them seems promising to explore. It may be beneficial to identify key biomarkers backward to better stratify patients receiving neoadjuvant immunotherapy.

Biological processes are dynamically altered depending on tumor burden and treatment. Tumor-associated immune cells, TME, or tumor neoantigens might change rapidly with tumor debulking under chemo-immunotherapy. Thus, dynamic biomarkers could be used to escalate or de-escalate preoperative therapeutic strategies. The development and validation of dynamic and easy-monitored biomarkers are less explored. A composite biomarker incorporating multiple other variables may be a novel direction for future research.

## Author contributions

Conception and design: YW, SH, and ZH. Administrative support: ZZ and ZH. Collection and assembly of reference: XF, WX, RL, and QZ. Graph drawing: YW and SH. Manuscript writing: all authors. All authors contributed to the article and approved the submitted version.
